# Dual Cross-Linked Chitosan/PVA Hydrogels Containing Silver Nanoparticles with Antimicrobial Properties

**DOI:** 10.3390/pharmaceutics13091461

**Published:** 2021-09-13

**Authors:** Dana M. Suflet, Irina Popescu, Irina M. Pelin, Daniela L. Ichim, Oana M. Daraba, Marieta Constantin, Gheorghe Fundueanu

**Affiliations:** 1 Petru Poni Institute of Macromolecular Chemistry, Aleea Grigore Ghica Voda 41A, 700487 Iasi, Romania; ipopescu@icmpp.ro (I.P.); impelin@icmpp.ro (I.M.P.); ghefun@icmpp.ro (G.F.); 2 Faculty of Medical Dentistry, “Apollonia” University of Iasi, 2 Muzicii Str., 700511 Iasi, Romania; danielaluminitaichim@yahoo.com (D.L.I.); oana_daraba@yahoo.com (O.M.D.)

**Keywords:** chitosan, poly(vinyl alcohol), hydrogels, silver nanoparticles

## Abstract

Stable chitosan/PVA-based hydrogels were obtained by combining covalent and physical cross-linking methods. As covalent cross-linkers, epoxy agents with different chain lengths were used, while freeze–thaw cycles were applied for additional physical cross-linking. The chemical structure of the hydrogel was examined by FTIR spectroscopy whereas the morphology was analyzed by SEM, showing well-defined pores with dimensions of around 50 μm in diameter. It was proved that gel fraction and the network morphology were deeply influenced by the synthesis conditions. Chitosan/PVA hydrogel showed a relative high swelling rate, reaching equilibrium in the first hour. The values obtained for the elastic modulus were relatively low (3–30 kPa); as a result, these hydrogels are soft and very flexible, and are ideal candidates for medical applications as wound or oral dressings. In addition, the natural antimicrobial activity of chitosan was enhanced by in situ generation of silver nanoparticles (AgNPs) under UV irradiation. The total amount of Ag from hydrogel was determined by elemental analyses and its crystalline state was confirmed by XRD. The CS/PVA hydrogels entrapped with AgNPs exhibited high inhibitory activity against *S. aureus* and *K. pneumonia*. The vitality tests confirmed the lack of cytotoxicity of CS/PVA hydrogels without and with AgNPs.

## 1. Introduction

Bacterial infections caused by various pathogens, for example *Staphylococcus aureus* (*S. aureus*), *Escherichia coli* (*E. coli*), *Klebsiella pneumonia* (*K. pneumonia*) or *Pseudomonas aeruginosa* (*P. aeruginosa*), still represent a major health problem. The healing time can be influenced by different factors such as genetic vulnerability, nutritional status, age, coexisting illnesses, the time of organism exposure to bacterial agents, excessive drugs intake, and air pollution [1]. Effective treatment consists of antibiotics administration, but an increasing body resistance or unwanted secondary effects have been observed, and for this reason, new therapeutic devices or drug delivery systems are being developed [2]. For treating infected skin wounds, oral or intestinal mucosal diseases, polysaccharide-based hydrogels with prolonged antimicrobial activity are frequently used [3]. Hydrogels can be impregnated with antibiotics, antimicrobial agents, and silver or gold nanoparticles, acting through non-stereospecific mechanisms that involve bacterial membrane disruption or biofilm generation [3,4].

Hydrogels are usually polymeric networks, extensively developed as intelligent systems for biomedical and pharmaceutical applications, including contact lenses [5], vascular prostheses and coatings for stents and catheters [6], tissue engineering [5], diagnostics [7], and drug delivery systems [8]. Hydrogels are hydrophilic networks capable of retaining large amounts of water yet remaining insoluble and maintaining their three-dimensional structure [9]. The polymers used to prepare hydrogels need to be physically and/or chemically cross-linked to prevent their dissolution. By chemical cross-linking, the polymer chains are strongly bonded via a covalent linkage, while the hydrogels which are physically cross-linked possess weak linkages (physical domain junctions, hydrogen bondings, hydrophobic interactions, ionic interactions, van der Waals interactions, or combinations of these [10,11,12]).

Natural and synthetic polymers offer many possibilities for the design and development of hydrogels that can be used in biomedical applications. However, their combination allows us to obtain ideal materials since they often cumulate synergistically in the properties of the two components. Among natural polymers, polysaccharides are frequently used in the manufacture of hydrogels, due to their non-toxic, biocompatible, and biodegradable properties. Many studies regarding the use of polysaccharide-based hydrogels as drug delivery systems [13], scaffolds [14], cartilage substitutes [15], and sorbent materials [16] have been reported in the literature. The most widely used polysaccharides for obtaining hydrogels with applications in medicine and pharmacy are cellulose, alginate, chitosan, starch, dextran, pullulan, and their derivatives [17,18].

Among them, chitosan (CS) is a cationic polysaccharide widely used in pharmaceutical formulations or for other biomedical applications [14,15,16,19,20,21,22,23]. Many researchers have highlighted its attractive proprieties, such as biodegradability, biocompatibility, cellular binding capability, antimicrobial [24], antifungal [25], antioxidant [26], antitumor activity [27], and wound healing properties [28]. These and other positive features, such as hydrophilic and cationic charges, make chitosan a suitable polymer for the delivery of active ingredients such as drugs, growth factors, stem cells, and peptides.

Regarding synthetic polymers, poly(vinyl alcohol) (PVA) is the most used of them for the synthesis the hydrogels with biomedical and pharmaceutical applications due to its high biocompatibility, non-toxicity, and non-carcinogenicity [29,30]. Studies on using PVA-based hydrogels or mixtures with natural/synthetic polymers are frequently reported in the literature with applications such as wound dressings [29], artificial articular cartilage [31], and drug delivery systems [32]. PVA-based hydrogels can be prepared by chemical or physical cross-linking. Physically cross-linked hydrogels (obtained by the freeze–thawing (F–T) technique) have drawn greater research attention [33,34]. Single or more cycles of the F–T for a concentrated PVA solution leads to the formation of a network without cross-linking agents. However, PVA-based hydrogels obtained by the F–T method have a low resistance when they are stored for a long time at room temperature [29]. In addition, PVA-based hydrogels possess insufficient elasticity and limited hydrophilicity which restrict their use for biomedical applications. Therefore, combining PVA with natural polymers is a solution to induce specific mechanical properties, to increase the biocompatibility and bioactivity of the materials [29].

Several recent investigations have focused on the combination of CS with PVA in order to obtain soft, flexible, and swellable hydrogels by different methods for medical applications [35,36]. As has been stated, chitosan is known for its weak activity against a wide range of microorganisms, in which the most acceptable antimicrobial mechanism is found to include the presence of charged groups in the polymer backbone and their ionic interactions with bacteria wall constituents. Therefore, the addition of a supplementary antimicrobial agent in the cross-linked network of the hydrogel could substantially improve its antibacterial activity. However, the choice of the antibacterial agent is a very important factor. Various antibiotics are often used as antibacterial agents, but a common problem is the possibility to develop allergies or resistance, especially in long-term use. As a viable alternative, silver nanoparticles (AgNPs) exhibit strong inhibitory effects against a wide variety of microorganisms, such as Gram-positive and Gram-negative bacteria [37]. The advantages regarding the use of AgNPs compared with other formulations include the possibility of using lower concentrations of AgNPs to achieve similar antibacterial effects as other antimicrobial agents [38]. Reduction of silver ions in solution is one of the general methods for AgNP preparation. However, the presence of a residual reducing reagent used for nanoparticle syntheses such as citric acid, sodium borohydride, or other organic compounds may exhibit biological hazards. Therefore, the use of UV irradiation to reduce Ag^+^ ions embedded in a polymer matrix seems to be a more appropriate approach for the preparation of nanosystems for bioapplications [39].

Most studies reporting the synthesis of CS/PVA-based hydrogels, by various methods, use concentrated solutions of PVA (10% *w*/*v* or more) [40,41] and few reports present the production of hydrogels with semi-diluted PVA solutions (6–10%) [42,43]. Furthermore, from our knowledge, data concerning the synthesis of CS/PVA hydrogel combining two cross-linking procedures (physically by freeze–thawing method and covalently using an epoxy cross-linking agent) have not been reported in the literature.

In this context, this paper presents original results regarding a novel method for the synthesis of stable CS/PVA-based hydrogels by a double cross-linking procedure: physically by a freeze–thawing method and covalently using an epoxy cross-linking agent. The morphology of new CS/PVA hydrogels was analyzed by scanning electron microscopy (SEM) and their chemical structure was confirmed by FTIR spectroscopy. The main characteristics of these hydrogels were studied, such as the gel fraction, the degree of swelling in pure water or phosphate buffers, and the mechanical behavior. Then, the native antimicrobial activity of chitosan was enhanced by the in situ generation of AgNPs under UV irradiation. The total amount of Ag from CS/PVA-based hydrogels was determined by elemental analyses and the crystalline form of Ag was confirmed by X-ray diffraction (XRD). The cytotoxicity and in vitro antibacterial activity of the hydrogels with or without AgNPs were also investigated.

## 2. Materials and Methods

### 2.1. Materials

Chitosan (CS) and poly(vinyl alcohol) (PVA, Mowiol^®^ 20–98 with M_w_ = 125.000 g/mol and 98% hydrolysis degree) were purchased from Sigma Aldrich Chemie GmbH (Steinheim, Germany). The deacetylation degree of CS was 82.79% determined by NMR spectroscopy, and the molecular weight, M_v_ = 233.6 kDa, was calculated from viscometric measurements according to the method described in the literature [44]. Ethylene glycol diglycidyl ether (EGDGE), 1,4-butanediol diglycidyl ether (BDDGE), hydrochloric acid (HCl), and sodium hydroxide (NaOH) were purchased from Merck (Darmstadt, Germany), and silver nitrate (AgNO_3_) from Honeywell Fluka (Seelze, Germany). All reagents were used without further purification and all experiments were performed using distilled water.

### 2.2. Methods

#### 2.2.1. CS/PVA Hydrogel Preparation

First, the CS solutions (1 or 2%, *w*/*v*) were prepared by dissolving a proper amount of CS in 0.1 M HCl solution at room temperature (RT) while stirring overnight. Then, the pH of the CS solutions was adjusted to 5.0 by gradually adding 0.1–10 M of NaOH solution. PVA solutions (1 or 2%, *w*/*v*) were prepared by dissolving the polymer in water under continuous stirring at 80 °C for 4 h. Hydrogels (CS/PVA) were prepared by mixing CS and PVA solutions in different ratios so as to obtain 0.5:1, 1:1, and 1:0.5 weight ratios between partners in hydrogels. Then, a volume of cross-linking agent (EGDGE or BDDGE) was slowly added, to make a 1:1 or 1:2 stoichiometric ratio between the amine groups of CS and the epoxy groups of the cross-linking agent ([NH_2_]:[epoxy]). The mixtures were poured into Petri dishes (d = 5 cm) and stored for 24 or 48 h at RT, until the chemical cross-linking reaction of the polymer chains occurred. Then, the samples were subjected to a freezing process for 24 h at −20 °C until the physical cross-linking of the PVA chains took place. The time of the thawing process (*t*_T_) was maintained the same as the initial storing time: 24 h or 48 h at RT. The F–T process was repeated for 3 and 6 cycles in order to establish the optimum conditions for the synthesis of the highest performing hydrogel.

The overall encoding of the hydrogels is CS_x_/PVA_y_R_z_(*t*_T_) wherein CS denotes chitosan, PVA means poly(vinyl alcohol), R represents cross-linking agent where E is EGDGE and B is BDDGE, *x* is the concentration of the CS in hydrogel, *y* shows the PVA concentration, *z* is the [NH_2_]:[epoxy] ratio, and *t*_T_ is the time at RT, before each freeze step. For example, CS_0.5_/PVA_1_E_2_(24) means the hydrogel was obtained starting with 0.5% CS (*w*/*v*), 1% PVA (*w*/*v*), EGDGE as cross-linker with [NH_2_]:[epoxy] = 1:2 and 24 h holding time (equal with the thawing time).

#### 2.2.2. In Situ Generation of AgNPs within Hydrogels

Briefly, AgNPs were generated in situ by immersing circular discs of hydrogel overnight (diameter = 10 mm, thickness = 1.5 mm) in the silver nitrate solutions with various concentrations (1 mM, 5 mM, and 10 mM). The silver ion-loaded discs were rinsed with distilled water (2 × 20 mL) in order to remove the excess of silver ions, then were exposed for 15 min to ultraviolet light using a UV lamp (365 nm; 9 W) for reducing the Ag^+^ to Ag^0^. Finally, the discs were freeze-dried for further characterization or kept in hydrated form for antimicrobial assays.

#### 2.2.3. Physico-Chemical Characterization of Hydrogels

##### Fourier-Transform Infrared Spectroscopy (FTIR)

FTIR analyses were used to ascertain the occurrence of covalent cross-links during the fabrication of the hydrogels. The FTIR spectra were recorded on KBr pellet using a Vertex 70 Bruker spectrometer (Bruker Optik GmbH, Ettlingen, Germany).

##### Scanning Electron Microscopy (SEM)

The surface and internal morphology of CS/PVA hydrogels were investigated using a Quanta 200 Scanning Electron Microscope (FEI Company, Bruno, Czech Republic) and a Verios G4 UC Scanning Electron Microscope (Thermo Scientific, Bruno, Czech Republic) equipped with an energy dispersive X-ray spectrometer (EDS, EDAX Octane Elect Super SDD detector, Ametek, Mahwah, NJ, USA) with a better magnification that allowed the visualization of AgNPs. The samples were coated with 10 nm platinum using a Leica EM ACE200 Sputter coater to provide electrical conductivity and to prevent charge buildup during exposure to the electron beam.

##### Gel Fraction

The gel fraction was used to evaluate the percentage of polymers (CS and PVA) involved in the covalent and physical cross-linking reaction. Briefly, after the last freezing cycle, the CS/PVA hydrogels were freeze-dried and weighed (*W_i_*, g). Then, to remove the non-cross-linked polymer chains and unreacted cross-linking agent, the hydrogels were washed with distilled water until the conductivity of the distilled water was 5 μS/cm^2^. Finally, the washed hydrogels were lyophilized again and weighed (*W_p_*, g). The gel fraction (*GF*, %) that can be assimilated with a degree of cross-linking, was calculated according to Equation (1) [45]:

(1) $$GF\left(\%\right)=\frac{W_{p}}{W_{i}}\times 100$$

where *W_p_* is the weight of purified sample in the dried state; *W_i_* is the weight of dried sample before purification.

##### Swelling Behavior

The swelling kinetic in aqueous medium was studied by the gravimetric method. Briefly, freeze-dried and weighed hydrogels (*W_d_*, g) were immersed in distilled water, in aqueous solutions with various pH (adjusted with 0.1 N NaOH or 0.1 N HCl) or in phosphate buffer solution (PBS) with pH = 6.8, simulating the oral environment. At selected time intervals, the hydrated hydrogels were carefully withdrawn, the excess of superficial water was removed with a wet filter paper, then the samples were weighed (*W_t_*, g). This procedure was repeated until no further weight increment occurred and equilibrium was attained (*W_e_*, g). The swelling ratio (*SR*) and equilibrium swelling ratio (*SR_eq_*) were calculated according to Equations (2) and (3), respectively:

(2) $$SR\left(\%\right)=\frac{\left(W_{t}-W_{d}\right)}{W_{d}}\times 100$$

(3) $$SR_{eq}\left(\%\right)=\frac{\left(W_{e}-W_{d}\right)}{W_{d}}\times 100$$

##### Mechanical Properties

Uniaxial compression tests were performed using a Texture Analyser (Brookfield Texture PRO CT3^®^, Brookfield Engineering Laboratories Inc., Middleboro, MA, USA) at RT. The hydrogels in the hydrated state with cylindrical shape (10–15 mm diameter and 6–3 mm height) were compressed with 0.067 N trigger load between two parallel plates with a compression rate of 0.1 mm/s up to 70% strain. The elastic modulus (*E*) was calculated according to Equation (4) [46]:

(4) E=σε=FAΔll0

where *σ* is the compressive stress (N/m^2^), *ε* is the strain, *F* is the force (N), *A* is the cross-sectional area of the hydrogel (m^2^), Δ*l* is the change in length, and *l*_0_ is the original length. The elastic modulus *E* was calculated from the slope of stress−strain curves between 5 and 15% compressions.

##### X-ray Diffraction (XRD)

The X-ray diffraction analysis of CS/PVA hydrogels with AgNPs was performed with a Rigaku Miniflex 600 diffractometer (Rigaku, Tokyo, Japan) using CuKα- emission (λ = 1.5406 Å) in the angular range (2θ) of 10° to 90°, at a scanning step of 0.01° and a recording rate of 5°/min. Background subtraction, smoothing and WPPF (Whole Powder Pattern Fitting) refinement of XRD data were made using the SmartLab II v.4 software package for powder X-ray diffraction analysis, while the diffraction peaks were identified using the Crystallography Open Database (COD).

##### Elemental Analyses

The amount of silver in hydrogels was determined using an atomic absorption spectrometer (ContrAA 800 D, Analytik Jena AG, Jena, Germany). The 5–10 mg of CS/PVA hydrogel loaded with AgNPs was digested in 1 mL of 65% nitric acid (HNO_3_) at ambient temperature for 24 h. Later, 1 mL of 65% HNO_3_ was added for a total digestion.

##### Antimicrobial Activity Assessments

The antimicrobial activities of the CS/PVA hydrogels without and with AgNPs were determined by the disk diffusion technique. The tests were conducted against typical reference strains of Gram-positive bacteria such as *S. aureus* ATCC 25923, and Gram-negative bacteria such as *E. coli* ATCC 11775, *K. pneumoniae* ATCC BAA–1705, and *P. aeruginosa* ATCC 10145. Disk diffusion test was performed on commercially available antimicrobial disks with MacConkey agar for *E. coli*, *P. aeruginosa*, and *K. pneumoniae*, and mannitol salt agar for *S. aureus*. A suspension of microorganisms of 0.5 McFarland density was inoculated on a plate with the substrate. The CS/PVA hydrogel discs with 12 mm diameter (d_s_, mm) were firstly hydrated in sterile deionized water and then placed on the agar plate and incubated for 24 h at 37 °C. The antibacterial activities were assessed by measurement of the diameters of the inhibition (d_in_, mm) zones according to the Kirby–Bauer method [47]. The experiments were repeated three times.

##### Cytotoxicity Assay

The cytotoxicity analysis of the hydrogels was performed using Human Dermal Fibroblasts adult (HDFa). The cells were grown in DMEM (Dulbecco’s Modified Eagle Medium, Life Technologies Limited., Paisley, UK) supplemented with 10% FBS (Fetal Bovine Serum) and an antibiotic cocktail consisting of 1% (*v*/*v*) penicillin–streptomycin and 1% (*v*/*v*) non-essential amino acids. The medium was changed every day. The cell culture flasks (NuncTM EasYFlask TM, ThermoFisher Scientific, ThermoFisher Scientific, Roskilde, Denmark) were incubated at 37 °C in a 95% humidified atmosphere and 5% CO_2_ (MCO-5AC CO_2_ Incubator, Panasonic Healthcare Co., Ltd., Sakata Oizumi-Machi Ora-Gun Gunma, Japan). Cells were allowed to grow in four culture flasks to reach 80% confluence and then were trypsinized with 0.25% trypsin solution at 37 °C for 3 min, followed by the addition of fresh medium to neutralize trypsin. Concentration of cells was 1 × 10^4^ cells/cm^2^. After centrifugation (Rotofix-32A, Hettich, Andreas GmbH Hettich & Co.KG, Tuttlingen, Germany,) and re-suspension in fresh medium, the viable cells were plated in flasks and allowed to settle and attach for 24 h prior to treatment. The sterilized hydrogels were incubated for 24 h in direct contact with the subculture cells. After the incubation time, the cell viability was determined using 3-(4,5-Dimethyl-2-thia zolyl)-2,5-diphenyl-2H-tetrazolium bromide (Merck Millipore, Darmstadt, Germany) following the MTT assay. Each type of hydrogel was tested in triplicate.

##### Statistical Analysis

Data were presented as mean ±SD. The ANOVA single factor was performed for statistical analysis and *p* < 0.05 was considered to be statistically significant.

## 3. Results and Discussions

### 3.1. Synthesis of CS/PVA Hydrogel

The new CS/PVA-based hydrogels were successfully obtained, from diluted solutions of PVA, by combining two cross-linking techniques: (a) in the first stage, the covalent cross-linking of the CS chains was performed at RT with EGDGE or BDDGE, known to have low toxicity [48,49]; (b) in the second stage, the physical cross-linking of the PVA chains was achieved by the freeze–thaw cycles [33]. With the aim to obtain a hydrogel with optimal properties, the influence of synthesis parameters on the hydrogel characteristics was studied (Table 1). Thereby, the weight ratio between CS and PVA, the length of the cross-linking agent, the [NH_2_]:[epoxy] ratio, the storage time at RT, and the number of freeze–thaw cycles were varied. The efficiency of the cross-linking process is well expressed by the gel fraction presented in Table 1.

*GF* values between 38 and 84% were obtained (Table 1), values that were deeply influenced by the synthesis conditions. Therefore, as a first general rule, the *GF* values increase with increasing the number of F–T cycles; more microcrystalline domains between polymer chains are formed, resulting in the increase of cross-linking density [33,50,51]. It must be noticed that a number of fewer than three F–T cycles led to poorly stable hydrogels that were difficult to handle due to a low degree of cross-linking (a low number of chemical and physical bonds). As a second general rule, the *GF* values increase with increasing the holding time (*t*_T_) at RT. In fact, maintaining the hydrogel for a longer period of time at room temperature inevitably leads to an increase in the degree of cross-linking by covalent bonds. Regarding the composition of the hydrogel, it can be observed that the maximum *GF* value of 84% was obtained for the hydrogels with the highest CS content and using EGDGE as a cross-linking agent. Covalent linkages between amino groups of CS and epoxy groups of cross-linker secure the chemical stability of the hydrogel. Moreover, EGDGE is more reactive than BDDGE in reaction with CS.

**Figure 1:**
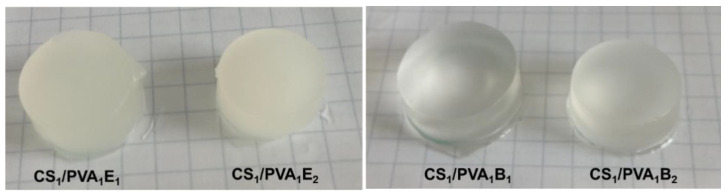
Optical photomicrographs of CS/PVA hydrogels after the last six freeze–thaw cycles and a holding time of 24 h.

Figure 1 shows optical photographs of the CS/PVA hydrogels in hydrated state after six F–T cycles and a holding time of 24 h. It is known that during the F–T process in the structure of the PVA-based hydrogel, two regions are created. These regions include areas with high density of PVA chains and areas with low density. Repeating the F–T process leads to the formation of crystallites in the PVA-rich area, which induces a randomly distributed heterogeneity in the structure, creating cloudy/opaque PVA hydrogels [52]. In our case, the transparent CS/PVA hydrogels were obtained by the covalent cross-linking with BDDGE, while the EGDGE led to opaque hydrogels. The transparency is probably caused by the longer length and higher hydrophobicity of BDDGE chains, compared with EGDGE, which prevents the formation of PVA densely crystalline areas.

### 3.2. Fourier-Transform Infrared Spectroscopy

**Figure 2:**
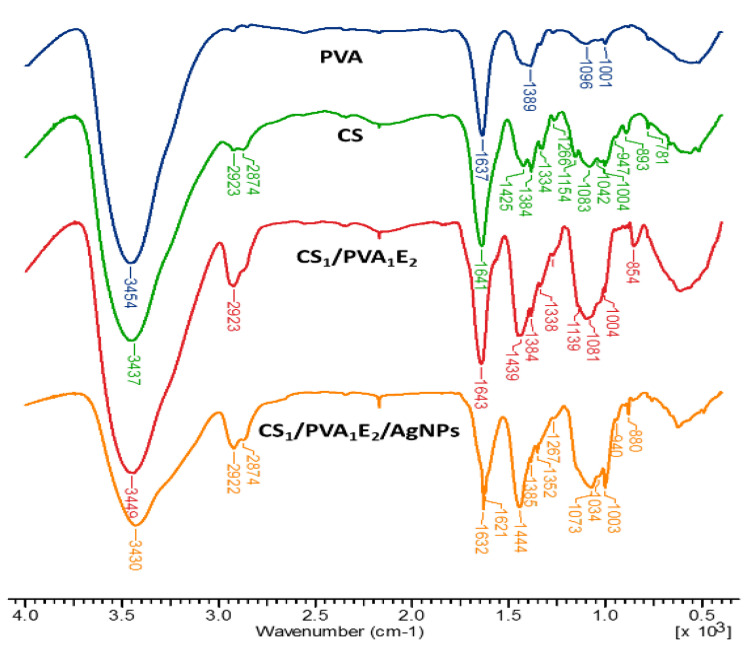
FT–IR spectra of chitosan (CS), poly(vinyl alcohol) (PVA), cross-linked hydrogels (CS_1_/PVA_1_E_2_), and hydrogel loaded with silver nanoparticles (CS_1_/PVA_1_E_2_/AgNPs).

The chemical structure of the CS/PVA hydrogels was assessed by FTIR spectrometry (Figure 2). FTIR spectra of hydrogels show both characteristic bands for parent polymers (CS and PVA) and the increase of intensity of some bands due to the presence of the cross-linking agent. Thus, the large band at 3437–3454 cm^−1^ is attributed to O–H and N–H stretching, as well as the intra- and inter-molecular hydrogen bonds, the bands at 2923–2873 cm^−1^ to C–H asymmetric and symmetric stretching from CH_2_ and CH groups, the band at 1637–1644 cm^−1^ is assigned to the associated water, C–OH from the glycosidic units of polysaccharide chains and also the presence of residual N-acetyl groups (C=O stretching of amide I), and N‒H in plane deformation coupled with C–N stretching of amide II (secondary amide) from CS. The bands from 1425–1444 cm^−1^ correspond to CH_2_ deformation groups, at 1384 cm^−1^ to –CH_3_ symmetric deformation, and at 1334–1338 cm^−1^ to C‒N bond stretching of amide III. The band at 1154 cm^−1^ was attributed to the glycosidic linkage (asymmetric bridge stretch) and the absorption bands in the range 1083–883 cm^−1^ belong to the C–O–C glycosidic ring (skeletal vibration involving the C–O stretch).

The increase in the intensity of the bands from 2923 cm^−1^ (CH_2_ groups), 1450–1437 cm^−1^ (CH_2_ bonds) and at 854–853 cm^−1^ (NH groups) in the spectrum of the hydrogel confirms the covalent cross-linking. In the case of hydrogel with AgNPs, where neutral Ag atoms (Ag^0^) form coordination bonds with NH_2_ and OH electron-rich groups, the FTIR spectrum presents a decrease in intensity of the band from 3430 cm^−1^, indicating that the N‒H vibrations were affected by the attachment of silver to the nitrogen from CS. The shift of the bands from 1643‒1004 cm^−1^ to higher or lower wavelengths or sometimes splitting (as the band from 1643 cm^−1^ was split in 1623 and 1621 cm^−1^) also reflects the interactions between Ag, O, and N atoms [53,54].

### 3.3. SEM Analyses

**Figure 3:**
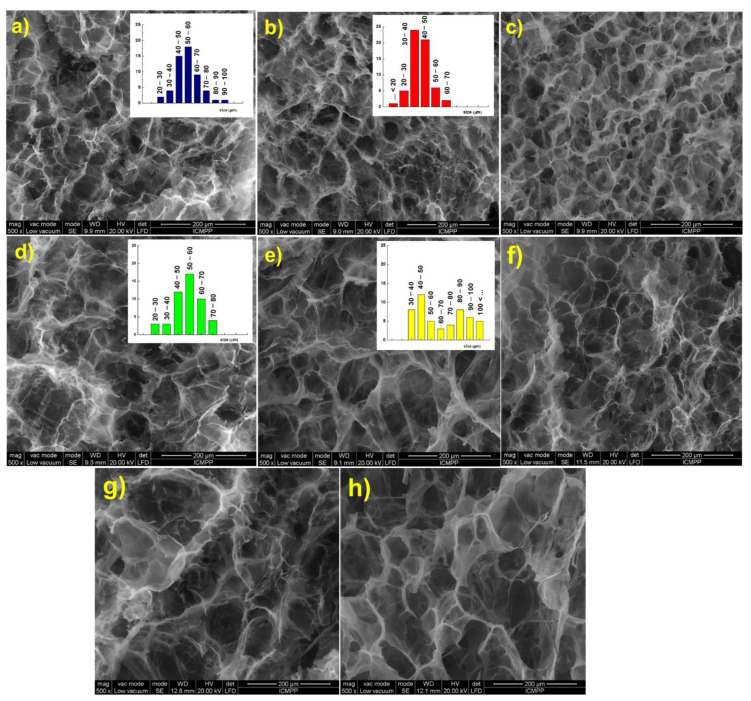
Scanning electron micrographs of the CS/PVA hydrogels in cross section: (**a**) CS_1_/PVA_1_E_1_(24); (**b**) CS_1_/PVA_1_E_2_(24); (**c**) CS_1_/PVA_1_E_2_(48); (**d**) CS_1_/PVA_1_B_1_(24); (**e**) CS_1_/PVA_1_B_2_(24); (**f**) CS_1_/PVA_1_B_2_(48); (**g**) CS_0.5_/PVA_1_E_2_(24); (**h**) CS_1_/PVA_0.5_E_2_(24).

Scanning electron micrographs of the hydrogels in cross section show a three-dimensional network with well-defined pores with a diameter ranging between 40 and 60 μm. As expected, the increase of [NH_2_]:[epoxy] molar ratio led to a decrease of both size and size distribution, due to the increasing number of cross-links between polymeric chains (Figure 3a,b). Furthermore, the increase of the holding time at RT from 24 h (Figure 3b) to 48 h (Figure 3c) led to a structure with smaller pores and thicker walls, when EGDGE was used as cross-linker. If BDDGE was used as cross-linker, the morphology of the hydrogel was not significantly influenced by the holding time (Figure 3e,f).

An influence of the length of cross-linking agent on the pore size and pore size distribution was observed. Thus, EGDGE, which possesses a short chain between the epoxy groups (two C atoms) led to a structure with smaller pores (Figure 3a–c), while BDDGE, with four C atoms between the epoxy groups, led to a structure with larger but irregular pores (Figure 3d–f). It must also be mentioned that an increase of the CS:PVA weight ratio from 0.5:1 (Figure 3g) to 1:1 (Figure 3b), and also the CS content in hydrogel, 1:0.5 ratio (Figure 3h), determined the generation of larger pores that follow an orderly arrangement.

### 3.4. Swelling Behaviour

If the hydrogel is synthesized as a biomaterial with potential applications in skin (or oral) dressing, it is absolutely necessary to determine the retention capacity and retention kinetics in water and simulated physiological conditions.

The retention capacity of water and PBS (pH = 6.8) at equilibrium is presented in Table 1. As is clearly evident, the hydrogels retain a large amount of water or PBS due to the hydrophilic nature of the two polymers. For example, the sample CS_1_/PVA_0.5_B_1_ obtained at a holding time of 24 h retains about 92-times more water and 74-times more PBS than its own weight in the dried state. The lower retention capacity of PBS than water, and this is noted for all samples, is due to the salting-out effect of the ions present in buffer that hinder the hydration of polymeric chains. The water retention capacity also depends on the holding time; the hydrogels obtained after 48 h retained less water or PBS than those obtained after 24 h as a result of the increasing number of hydrogen and covalent bonds between polymer chains that makes the network less expandable. The length of the cross-linking agent as well as the [NH_2_]:[epoxy] molar ratio deeply influenced the retention capacity of the CS/PVA hydrogels. Hydrogels cross-linked with BDDGE (4 C atoms between epoxy groups) have higher values of solvent retention than those obtained with EGDGE (2 C atoms between epoxy groups). A longer cross-linker leads to a hydrogel with larger pores and therefore with a higher water (PBS) retention capacity.

**Figure 4:**
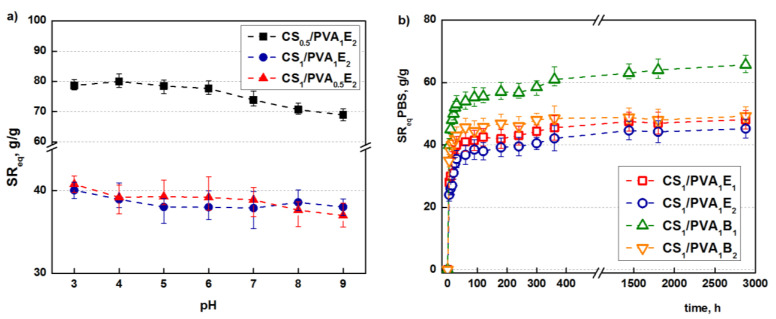
Influence of the pH of the swelling medium on the *SR*_eq_ (*t*_T_ = 24 h) (**a**). Swelling kinetics in simulated physiological conditions, PBS at pH = 6.8 (*t*_T_ = 24 h) (**b**).

Concerning the [NH_2_]:[epoxy] molar ratio, it is obvious that a higher ratio of cross-linker compared to the amino groups leads to a more efficient cross-linking reaction and therefore to a less expandable structure that retains less water (PBS). Because CS/PVA hydrogel contain ionizable amino groups with a pK_a_ = 6.5, the influence of pH on the swelling capacity was studied [55]. As shown in Figure 4a, the highest values of swelling ratios are obtained at the lowest values of pH due to the full protonation of the amine groups which induces electrostatic repulsions and structure expansion [56], and then the swelling ratio decreases. However, because a significant part of the primary amine groups is transformed into secondary amines during the covalent cross-linking, the influence of pH is not significant.

Besides the retention capacity, an important characteristic of a hydrogel used in biomedical applications is the swelling rate until it reaches equilibrium. Tanaka and co-workers proved that the promptness of the swelling response is inversely related to the size of the hydrogel [57]. Furthermore, a porous structure facilitates the migration of water through the pores (“convective” transport) of the hydrogels, increasing the swelling rate [58].

The hydrogels synthesized in this study have relatively small sizes and porous structures. As follows, they display relatively high swelling rates; in particular, after 1 h, equilibrium is reached (Figure 4b).

### 3.5. Mechanical Properties

**Figure 5:**
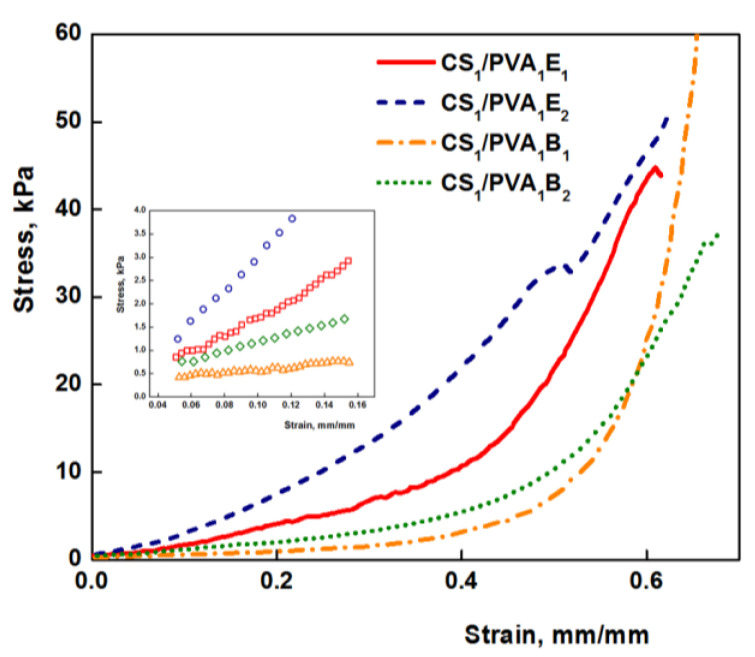
Stress–strain curve for CS/PVA hydrogel under compressive loading (*t*_T_ = 24 h). In detail the linear range for determining the elastic modulus is represented.

The mechanical properties of hydrogels are decisive factors for their future biomedical applications. Compression tests were performed to get information about the elastic behavior of the hydrogels (Figure 5). The values of elastic modulus were calculated between 5 and 15% compression, where the stress–strain curves are linear (Figure 5, inset), and the obtained values are presented in Table 1. Values of the elastic modulus cover a wide range from 2.58 kPa for sample CS_0.5_/PVA_1_B_1_ to 31.70 kPa for sample CS_1_/PVA_1_E_2_. As a general rule, the Young modulus increases with the increasing amount of cross-linker, due to the large number of the cross-links between polymeric chains and therefore a reduced mobility of the macromolecules. The increase of the number of carbon atoms of the cross-linker from 6 to 8 determined a decrease of the elastic modulus. Firstly, the longer-length cross-linker (BDDGE) has a lower reactivity than the shorter-length cross-linker (EGDGE) and gives rise to less cross-linked and therefore soft and more elastic three-dimensional structures. Secondly, a longer length of the cross-linker leads to an increased chain mobility and therefore elasticity. The increase of CS/PVA weight ratio from 0.5:1 to 1:1 (CS_0.5_/PVA_1_ to CS_1_/PVA_1_) determined a significant increase of the compression modulus due to the increased number of covalent cross-links, resulting a stiffening of the hydrogels. In contrast, the decrease of the PVA amount in hydrogels from CS_1_/PVA_1_ to CS_1_/PVA_0.5_ led to softer materials. These results are confirmed by the literature data showing that PVA can be used as a polymer for reinforcing hydrogels [29].

However, the values of the modulus of elasticity of hydrogels are relatively low (3–30 kPa) compared to other polymeric materials, which makes them very elastic. As a result, these hydrogels are ideal candidates for medical applications, as wound or oral dressings that must be strong, soft, and flexible materials [59,60]. Nevertheless, hydrogels with very low values of Young modulus are less resistant, and, therefore, for subsequent experiments samples with average values were chosen (CS_1_/PVA_0.5_E_2_ and CS_1_/PVA_0.5_B_2_ (*t*_T_ = 24 h)).

### 3.6. In Situ Synthesis and Immobilization of Silver Nanoparticles

Chitosan is known to exhibit antibacterial activity, but this activity may be diminished in mixtures with other compounds [61]. Thus, to improve the antimicrobial effect, CS/PVA-based hydrogels were immersed in AgNO_3_ solutions of different concentrations. The reduction of Ag^+^ ions to Ag^0^ was performed in the presence of UV radiation as an ecological method that avoids the use of potentially toxic reducing agents. In addition, this method can allow the sterilization of hydrogels at the same time. In this context, the effect of AgNO_3_ solution concentration on silver particle size and biological properties was studied.

**Figure 6:**
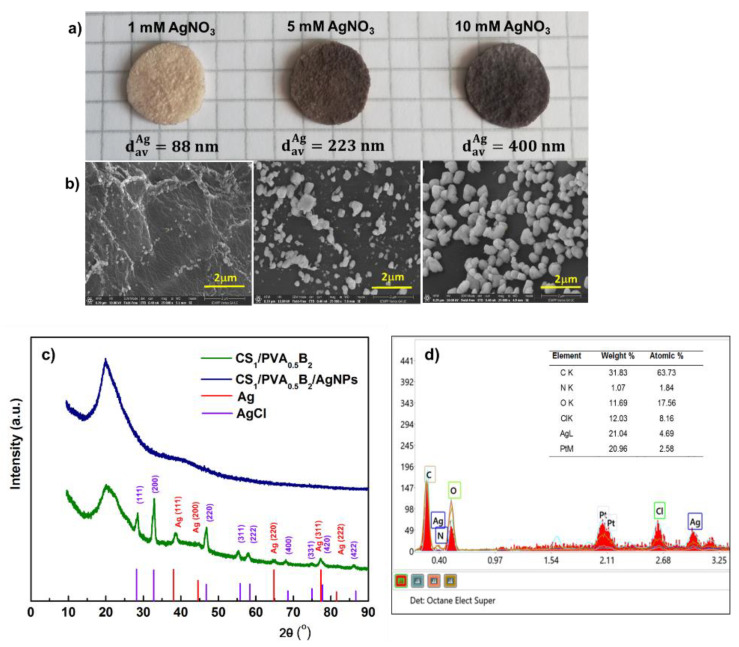
Optical microphotographs of CS/PVA hydrogel (sample CS_1_/PVA_0.5_B_2_ (*t*_T_ = 24 h)), in situ loaded with silver nanoparticles by using different concentrations of AgNO_3_ (**a**). Scanning electron micrographs (cross-sections) of the corresponding hydrogels loaded with Ag nanoparticles (**b**). XRD patterns of hydrogel without and with AgNPs after immersion in 10 mM AgNO_3_ (**c**). Energy-dispersed X-ray spectrometry (EDS) patterns of the hydrogel with AgNPs after immersion in 10 mM AgNO_3_ (**d**).

The SEM images showed that the size of the Ag particles increases from 88 nm to 400 nm (average diameter, d_av_) (*p* < 0.05) with the increase of AgNO_3_ concentration from 1 mM to 10 mM (Figure 6a,b). This aspect was supported by the elemental analysis of Ag from hydrogel. Thus, we determined a content of 0.11, 1.1, and 2.3 wt.% Ag in hydrogel after immersion in 1 mM, 5 mM, and 10 mM AgNO_3_, respectively.

The crystalline structure of the AgNPs was analyzed by XRD. The XRD spectrum of CS/PVA hydrogel (Figure 6c) exhibited a major peak at 20.2° which corresponds to the polymorph of the pattern [62] and certifies that a strong interaction occurred between CS and PVA molecules in the hydrogel [63]. The spectrum of CS/PVA hydrogel with AgNPs shows the diffraction peaks at around 38.1°, 44.3°, 64.4°, 77.3°, and 81.5° which were associated to the (111), (200), (220), (311), and (222) crystalline planes of face-centered cubic crystals of the metallic Ag structure in hydrogel, which confirms the successful synthesis of crystalline AgNPs (space group 225:Fm-3m, Card No. 9008459) [64]. In addition, the spectrum showed the presence, together with Ag^0^, of AgCl nanoparticles resulting from the reaction of Ag^+^ with the counterion Cl^-^ from the ionized form of amino groups of chitosan, due to solubilization of CS in HCl. Diffraction peaks observed at 28.2°, 32.6°, 46.9°, 55.63°, 58.3°, 68.5°, 75.6°, and 77.9° correspond to the face-centered cubic structures (111), (200), (220), (311), (222), (400), (331), and (400) planes of AgCl Nps, respectively [65]. These results were confirmed by energy dispersive spectroscopy (EDX); accordingly (Figure 6d), the elements C, O, N, Ag, and Cl were assigned to the sample and the Pt coating layer, respectively.

### 3.7. Antimicrobial Activity Assessments

**Figure 7:**
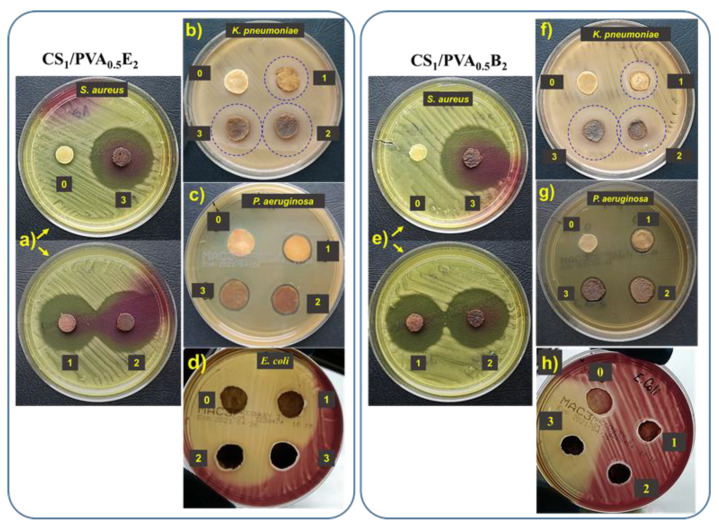
Antibacterial activity of CS_1_/PVA_0.5_E_2_ and CS_1_/PVA_0.5_B_2_ hydrogels against *S. aureus* (**a**,**e**), *K. pneumonia* (**b**,**f**), *P. aeruginosa* (**c**,**g**), and *E. coli* (**d**,**h**): zone 0, the hydrogel without AgNPs; zone 1, hydrogel with AgNPs obtained in 1 mM AgNO_3_ solution; zone 2, hydrogel with AgNPs obtained in 5 mM AgNO_3_ solution; zone 3, hydrogel with AgNPs obtained in 10 mM AgNO_3_ solution.

The antibacterial activity of CS/PVA hydrogels with and without AgNPs was investigated using Gram-negative (*E. coli*, *P. aeruginosa*, and *K. pneumoniae*) and Gram-positive (*S. aureus*) bacteria by the disk diffusion method. Regardless of the Ag percentage or the size of AgNPs, all hydrogels with silver nanoparticles showed intense inhibitory activity against *S. aureus* (Table 2, Figure 7a,e). In this case, the inhibitory zone diameter (d_iz_) was between 42 mm and 45 mm. We also observed an intense inhibitory activity against *K. pneumonia*. In this case, both the amount of Ag in the hydrogel and the AgNPs size had a slight influence on the inhibition of this bacterium, observing an increase in d_iz_ from 20 mm to 24 mm with increasing of Ag percentage and size (Figure 7b,f). The hydrogels loaded with AgNPs showed an insufficient activity against *P. aeruginosa* (Table 2, Figure 7c,g) and *E. coli* (Table 2, Figure 7d,h) when the d_iz_ was less than 15 mm. The hydrogels without AgNPs did not show inhibitory activity, probably due to the fact that the majority of amine groups in CS are involved in the cross-linking reactions. The results are not totally consistent with those obtained by Li et al. (2019) [66], since our AgNP-loaded hydrogels showed a strong antibacterial effect against *S. aureus* and *K. pneumoniae* and have almost no activity against *E. coli.* In fact, the antimicrobial activity was dependent on the content in AgNPs, increasing with the increase of Ag percentage in hydrogel, with this aspect largely demonstrated in the literature [24,29,38,54].

### 3.8. Viability Assay

Biocompatibility and the lack of cytotoxicity are imperative requirements when designing new wound or oral dressing materials. Silver nanoparticles and/or silver ions are known to be potentially toxic toward not only bacteria but also healthy, live cells [67].

**Figure 8:**
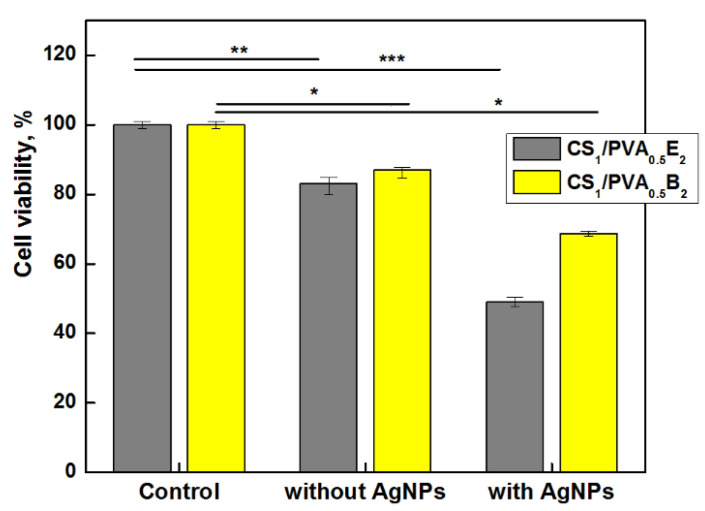
Cells viability of CS_1_/PVA_0.5_E_2_ and CS_1_/PVA_0.5_B_2_ hydrogels (*t*_T_ = 24 h) with and without AgNPs (1 mM AgNO_3_), after 24 h (* *p* < 0.001 ** *p* < 0.01, *** *p* < 0.05).

Taking into account these aspects, the vitality of dermal fibroblasts in direct contact with hydrogels with and without AgNPs was investigated. The results showed a slight decrease of cell vitality, 83–85% after 24 h, for CS_1_/PVA_0.5_ hydrogels without AgNPs (Figure 8). However, as was expected, the cell viability decreased in the presence of AgNPs, even for the samples with a small number of AgNPs and low amount of Ag in hydrogel. Nevertheless, the cell viability for CS_1_/PVA_0.5_B_2_ sample was 68%, which places the sample at the threshold of cytotoxicity and non-cytotoxicity. The hydrogel samples with higher content of AgNPs (>0.11 wt.%) and larger size (>100 nm) showed a cell viability smaller than 50% (data not shown) due to the higher concentration of released silver ions. Similar results were found by Li et al. [66] when they tested the growth of NIH3T3 cells in contact with AgCl NP-loaded hydrogels, a higher content of AgCl NPs (1.8 wt.%) in hydrogel causing a strong cytotoxic effect. In fact, the low toxicity reported by other researchers is due to the fact that they used another test approach, namely the extract method which does not involve direct contact between cells and nanoparticles [38,68]. We chose direct contact to test the skin’s reaction to a potential applicability of the hydrogel in skin dressing.

Generally, it is a contradiction between antimicrobial activity and cell viability, and therefore the optimal concentration of nanoparticles must be chosen to satisfy both requirements. Taking into account these considerations, the CS_1_/PVA_0.5_B_2_ sample loaded with 0.1 wt.% AgNPs smaller than 100 nm showed, at the same time, a good antimicrobial activity and an acceptable cytotoxicity.

## 4. Conclusions

New CS/PVA-based hydrogels were obtained using diluted PVA solutions by combining chemical and physical cross-linking methods. Two epoxy cross-linking agents with different chain lengths were used and the chemical reaction took place for 24 or 48 h. Then, the systems were subjected to F–T cycles in order to allow the formation of physical networks. The chemical structure was confirmed by FT-IR spectroscopy, and the morphology of the hydrogels was analyzed by SEM when well-defined pores having dimensions of 30–60 μm were observed. The gel fraction and the network morphology were influenced by the obtaining conditions. Hydrogels with the highest gel fractions were obtained at six F–T cycles, when the time at RT (when chemical cross-linking occurred) was 48 h, and the CS/PVA ratio was 1:0.5. The cross-linker agent with a longer chain, BDDE, confers a weaker cross-linking compared with EGDE, but at a lower cytotoxicity.

CS/PVA cryogels showed a fast swelling capacity when the equilibrium was reached in the first hour. The swelling degree in water and in PBS with pH = 6.8 was influenced by the chemical cross-linking: cross-linking agent, [epoxy]:[NH_2_] ratio, and *t*_T_. The mechanical tests showed an elastic behavior of hydrogels with low elastic modulus values and without cracking up to 70% compression.

The native antimicrobial activity of chitosan was enhanced by the in situ generations of silver nanoparticles under UV irradiation. The size of the AgNPs can be controlled by varying the concentration of AgNO_3_ solutions used to immerse the CS/PVA hydrogels prior to UV irradiation. The increase in the size of Ag particles with the increase of the concentration of the AgNO_3_ solution was proven by SEM and elemental analyses; AgNPs with dimensions of 88 nm AgNPs was obtained with a 1 mM AgNO_3_ solution. The XRD analysis highlighted a face-centered cubic crystal of metallic Ag structure in hydrogel that confirms the successful synthesis of crystalline AgNPs.

The hydrogels with entrapped AgNPs present a high inhibitory activity against *S. aureus* (Gram-positive bacteria) and *K. pneumonia,* but low activity for *P. aeruginosa* and *E. coli,* the last three being Gram-negative bacteria. The cell vitality test showed that the hydrogels without and with AgNPs smaller than 100 nm may be considered non-cytotoxic.

The swelling behavior, the mechanical properties, and the antimicrobial activity of these hydrogels recommend their use in the manufacture of antibacterial devices with biomedical applications.

## Figures and Tables

**Table 1:** The influence of synthesis parameters on the main characteristics of CS/PVA hydrogels.

Sample Code	Time (h)	GF, %		SR_eq_ *, g/g		Elastic Modulus **	
		3c	6c	Water	PBS 6.8	(kPa)	R^2^
CS_0.5_/PVA_1_E_1_	(24)	52.8 (±1.3)	61.6 (±1.4)	72 (±3)	67 (±4)	2.9 (±0.4)	0.9895
CS_0.5_/PVA_1_E_2_		58 (±2)	66.8 (±1.2)	64 (±2)	62 (±2)	6.1 (±0.3)	0.9906
CS_0.5_/PVA_1_B_1_		38 (±3)	45.8 (±1.3)	82 (±3)	75 (±2)	2.6 (±0.2)	0.9528
CS_0.5_/PVA_1_B_2_		43.3 (±1.2)	52.2 (±1.1)	79 (±3)	73 (±2)	9.8 (±0.4)	0.9874
CS_1_/PVA_1_E_1_		54.1 (±0.9)	68.5 (±1.2)	61 (±3)	48.1 (±1.1)	20.7 (±1.5)	0.9919
CS_1_/PVA_1_E_2_		58.2 (±1.2)	69 (±3)	48 (±3)	45.3 (±0.5)	27.4 (±0.5)	0.9942
CS_1_/PVA_1_B_1_		41.2 (±0.8)	57 (±2)	75 (±2)	65 (±2)	10.8 (±0.1)	0.9383
CS_1_/PVA_1_B_2_		56 (±2)	67 (±3)	69 (±2)	49 (±2)	15.1 (±0.6)	0.9908
CS_1_/PVA_0.5_E_1_		64 (±3)	78 (±5)	49 (±3)	44.9 (±1.4)	17.1 (±1.3)	0.9907
CS_1_/PVA_0.5_E_2_		69 (±4)	81 (±5)	46 (±3)	42.3 (±1.1)	18.7 (±1.5)	0.9954
CS_1_/PVA_0.5_B_1_		62 (±3)	74 (±5)	91 (±5)	74.0 (±1.1)	14.7 (±1.2)	0.9885
CS_1_/PVA_0.5_B_2_		62 (±3)	76 (±5)	69 (±3)	65.9 (±1.1)	14.6 (±1.4)	0.9278
CS_0.5_/PVA_1_E_1_	(48)	55 (±2)	70 (±4)	68 (±3)	65 (±3)	3.2 (±1.2)	0.9295
CS_0.5_/PVA_1_E_2_		60 (±2)	73 (±4)	61 (±3)	49 (±2)	4.8 (±1.2)	0.9774
CS_0.5_/PVA_1_B_1_		39.5 (±0.4)	71 (±4)	78 (±4)	83 (±3)	-	-
CS_0.5_/PVA_1_B_2_		45.9 (±1.4)	74 (±2)	75 (±4)	82 (±3)	2.9 (±0.4)	0.9292
CS_1_/PVA_1_E_1_		56 (±2)	74 (±2)	52 (±3)	48.1 (±1.1)	28.6 (±1.6)	0.9922
CS_1_/PVA_1_E_2_		62 (±2)	75 (±2)	44 (±2)	43 (±2)	31.7 (±1.1)	0.9934
CS_1_/PVA_1_B_1_		53.5 (±1.5)	63 (±2)	65 (±3)	61.7 (±1.2)	4.0 (±1.4)	0.9736
CS_1_/PVA_1_B_2_		60.4 (±1.1)	70 (±2)	61 (±3)	59.3 (±1.1)	7.2 (±0.9)	0.9821
CS_1_/PVA_0.5_E_1_		70 (±3)	77 (±3)	47 (±2)	42.5 (±1.4)	16.3 (±1.1)	0.9883
CS_1_/PVA_0.5_E_2_		71 (±3)	84 (±4)	44 (±2)	41 (±2)	16.6 (±1.4)	0.9856
CS_1_/PVA_0.5_B_1_		63 (±2)	76 (±4)	84 (±5)	69 (±3)	-	-
CS_1_/PVA_0.5_B_2_		69 (±3)	76 (±4)	63 (±4)	58 (±3)	6.7 (±1.6)	0.9904

*, **-SR_eq_ and Elastic modulus were determined for the hydrogels obtained after six freeze–thaw cycles.

**Table 2:** Antibacterial properties of the CS_1_/PVA_0.5_E_2_ and CS_1_/PVA_0.5_B_2_ hydrogels against *E. coli*, *P. aeruginosa*, *S. aureus*, and *K. pneumoniae*.

	Hydrogel	CS_1_/PVA_0.5_E_2_				CS_1_/PVA_0.5_B_2_			
Microorganism		Zone 0	Zone 1	Zone 2	Zone 3	Zone 0	Zone 1	Zone 2	Zone 3
*E. coli*		‒	‒	+	+	‒	‒	+	+
*P. aeruginosa*		‒	+	+	+	‒	+	+	+
*S. aureus*		‒	+++	+++	+++	‒	+++	+++	+++
*K. pneumoniae*		‒	++	++	++	‒	++	++	++

‘‒’ no effect; ‘+’for d_iz_ < 15 mm; ‘++’ for 15 mm < d_iz_ < 25 mm; ‘+++’ for d_iz_ > 25 mm.

## Data Availability

The data presented in this study are available on request from the corresponding author.
